# Distribution of the etiological factors and symptoms of true aneurysms of subclavian and axillary arteries

**DOI:** 10.1186/1749-8090-8-S1-P47

**Published:** 2013-09-11

**Authors:** O Gokalp, I Yurekli, L Yilik, U Yetkin, T Gunes, M Akyuz, B Ozcem, O Tetik, G Ilhan, A Gurbuz

**Affiliations:** 1 Izmir Katip Celebi University Ataturk Training and Research Hospital, Department of Cardiovascular Surgery, Izmir, Turkey

## Background

Isolated aneurysm of subclavian or axillary artery is very rarely seen.

## Methods

Eight patients that were operated on between February 1998 and December 2007 due to true aneurysms of the subclavian and axillary arteries were examined. Six of the patients (75%) were male. Median age was 58 (38-73). Two of these patients had subclavian and 6 of them had axillary artery aneurysms. Median diameter of the aneurysm was 46 mm (30-60).

## Results

Chief complaint was ischemic symptoms in 2 (25%) patients, non-disturbing mass in 3 (38%), feeling of compression in 2 (25%) and bleeding in 1 (12%) patient. Etiological factors include atherosclerosis in 5 patients (62%), a probable connective tissue disorder in 1 patient (13%) and idiopathic in 2 patients (25%). In these last 2 patients no atherosclerotic changes were prominent histopathologically and no other factors were identified; thus considered as idiopathic. One patient with a probable connective tissue disorder had no histopathological diagnosis. But he was phenotypically marfanoid and his past medical history was significant for surgery due to iliac artery aneurysm. Contrasted CT examination revealed no other aneurismatic segment preoperatively. Past medical history of another patient was also significant for surgery of contralateral axillary artery aneurysm. But histopathologic examination revealed atherosclerotic changes. Therefore this patient was not considered as having connective tissue disorder. One patient with ruptured axillary artery aneurysm was operated on emergently with a chief finding of bleeding.

## Conclusion

One of two cases with subclavian artery aneurysm that we operated on had acute ischemia findings of the upper extremity and the other had a non-disturbing mass. Consistent with the literature, 2 of our cases with axillary artery aneurysm had non-disturbing mass, 2 had feeling of compression, 1 had active bleeding and 1 had findings of acute ischemia.

